# *Lactobacillus plantarum* 06CC2 reduces hepatic cholesterol levels and modulates bile acid deconjugation in Balb/c mice fed a high‐cholesterol diet

**DOI:** 10.1002/fsn3.1909

**Published:** 2020-10-26

**Authors:** Masao Yamasaki, Mikako Minesaki, Asuka Iwakiri, Yuko Miyamoto, Kenjiro Ogawa, Kazuo Nishiyama, Chuluunbat Tsend‐Ayush, Tsendesuren Oyunsuren, Yiran Li, Tomoki Nakano, Masahiko Takeshita, Yuo Arima

**Affiliations:** ^1^ Graduate School of Agriculture University of Miyazaki Miyazaki Japan; ^2^ Organization for Promotion of Tenure Track University of Miyazaki Miyazaki Japan; ^3^ Food and Biotechnology School Mongolian University of Science and Technology Ulaanbaatar Mongolia; ^4^ Mongolian Biotechnology Association Ulaanbaatar Mongolia; ^5^ Research and Development Division Minami Nihon Rakuno Kyodo Co. Ltd. Miyakonojo Japan

**Keywords:** bile acid, butyrate, cholesterol, *Lactobacillus plantarum*, mice

## Abstract

Previous study suggested that dietary intake of *Lactobacillus plantarum* 06CC2 (LP06CC2) isolated from Mongolian dairy products showed various health beneficial effects. Here, the effect of LP06CC2 on the cholesterol metabolism in mice fed a cholesterol‐loaded diet was evaluated. Cholesterol and LP06CC2 were incorporated into the AIN93G‐based diet to evaluate the effect on cholesterol metabolism in Balb/c mice. Serum and liver cholesterol levels were significantly increased in mice fed a cholesterol‐loaded diet whereas the LP06CC2 ingestion suppressed the increase of liver cholesterol. LP06CC2 suppressed the increase of the hepatic damage indices. The increase of the cecal content and fecal butyrate were observed in mice fed LP06CC2. The analysis of bile acids clearly showed that LP06CC2 increased their deconjugation indicating the decrease of bile acid absorption. The protein expression of hepatic Cyp7A1 was also suppressed by LP06CC2 in mice fed cholesterol. Finally, in vitro studies showed that LP06CC2 had the most potent ability to deconjugate bile acids using glycocholate among the tested probiotic lactic acid bacteria isolated from Mongolian dairy products. Taken together, LP06CC2 is a promising microorganism for the reduction of the cholesterol pool via modulation of bile acid deconjugation.

## Graphical abstract

*Lactobacillus plantarum* 06CC2 (LP06CC2) is a Mongolian dairy product derived lactic acid bacteria. Ingestion of LP06CC2 decreased the liver cholesterol, hepatic damage, and increased fecal butyrate. LP06CC2 increased the deconjugation of bile acids to promote the excretion of bile acids. Together, LP06CC2 is a promising microorganism for the reduction of the cholesterol pool via modulation of bile acid deconjugation.

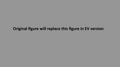

## 1 INTRODUCTION

Recent studies on the function of lactic acid bacteria (LAB) have suggested various kinds of health‐promoting effects of these microorganisms such as immunomodulation, anti‐inflammation, and anti‐metabolic disorder (Azad et al., 2018; Mazloom et al., 2019; Plaza‐Diaz et al., 2019). Actually, preventive effects against metabolic disorders such as the reduction of the body fat and blood uric acid have been applied to the functional foods in Japan based on scientific evidence. In addition, data showed the reduction of abnormal serum and liver lipid profiles indicating the putative usefulness of the LAB application for the prevention of lipid disorders and cardiovascular disease (Kurajo et al., 2018). Notably, the cholesterol‐lowering effect was produced by various LAB through different molecular mechanisms. Therefore, LAB administration is expected to be a promising way to prevent or improve the cholesterol metabolism‐related disorders. Among LAB, certain strains of *Lactobacillus plantarum* are shown to be useful for the reduction of the risk associated with cholesterol disorder in mice (Qu et al., 2020; Wang et al., 2019) and humans (Bukowska et al., 1998; Fuentes et al., 2013; Higashikawa et al., 2010). Several molecular mechanisms of this effect have been proposed as follows; assimilation of cholesterol (Dora & Glenn, 2002; Lim et al., 2017; Michael et al., 2016), promotion of bile acid or cholesterol excretion (Li et al., 2014; Qu et al., 2020; Zhai et al., 2019), and manipulation of hepatic cholesterol metabolism (Kim et al., 2014; Qu et al., 2020) In the context of these findings, it is of note that recent systematic study in *L. plantarum* showed ability to deconjugate bile acids (Prete et al., 2020). The apical sodium‐dependent bile acid transporter (ASBT)/ileal bile acid transporter (IBAT)/solute carrier family 10 member 2 (SLC10A2) is highly expressed in the small intestine, and most of the conjugated bile acids secreted into the gastrointestinal lumen are absorbed through these transporters (Aguiar Vallim et al., 2013). About 95% of the bile acids secreted into the gastrointestinal lumen are recycled by the enterohepatic circulation, and some bile acids enter the lower gastrointestinal tract. Bile salt hydrolase of the intestinal bacteria removes amino acids from the conjugated bile acids and they escape uptake by ASBT/SLC10A2, reducing the bile acid pool in the body (Pavlović et al., 2012). Our previous study showed that *L. plantarum* 06CC2 (LP06CC2) isolated from Mongolian dairy products was able to alleviate influenza infection (Takeda et al., 2011) and stimulate the induction of helper type‐1 T cells (Takeda et al., 2013). In contrast, the effect of LP06CC2 administration on the cholesterol metabolism has not been evaluated. The aim of this study was to evaluate the cholesterol‐lowering effect of LP06CC2 on mice fed a cholesterol‐loaded diet.

## 2 EXPERIMENTAL SECTION

### 2.1 Preparation of LP06CC2

*Lactobacillus plantarum* 06CC2 strain (LP06CC2), a potential probiotic from Mongolian dairy products, tolerates bile and gastric acids and adheres to Caco‐2 cells. LP06CC2 was cultured at 37°C for 18 hr in de Man, Rogosa, and Sharpe (MRS) broth (Merck). Microorganisms were harvested by centrifugation at 1,500 *g* for 5 min, washed twice with phosphate‐buffered saline, and lyophilized. The lyophilized LP06CC2 was stored at −30°C until the preparation of experimental diets.

### 2.2 Mice and diet

Studies were conducted using 5‐week‐old male Balb/c mice purchased from Japan SLC, Inc. and maintained at 22°C in a humidity‐controlled room with a 12‐hr light‐dark cycle. All mice were acclimatized for 1‐week and assigned to four groups (*n* = 6 each). Mice were fed the AIN‐93G‐based normal diet (ND), a high‐cholesterol diet (HCD) including 0.50% cholesterol and 0.25% sodium cholate, ND with the addition of LP06CC2 (5 wt%), or HCD with the addition of LP06CC2 (5%) for 3 weeks. Detailed compositions are shown in Table 1. Diets were vacuum‐packed and stored at 4°C. The mice had free access to the corresponding diet and water. The body weights and food intake were recorded every other day. The feces were collected from each mouse every other day and preserved at −30°C. The animal studies were conducted in accordance with the Guide for the Care and Use of Laboratory Animals of the University of Miyazaki (Animal Experiment Committee of University of Miyazaki: approval number 2017‐006) and in compliance with the Law Concerning the Protection and Control of Animals (Japan Law No. 105), Standards Relating to the Care and Management of Laboratory Animals and Relief of Pain (Notification no. 88 of the Ministry of the Environment, Japan), and The Guidelines for Animals Experimentation (the Japanese Association for Laboratory Animal Science). At the end of the feeding period, blood was collected from the heart in fasted condition under a triple anesthesia mix of 0.75 mg/kg medetomidine hydrochloride (Domitor; Nippon Zenyaku Kogyo Co., Ltd.), 4.0 mg/kg midazolam (Dormicum; Astellas Pharma Inc.), and 5.0 mg/kg butorphanol tartrate (Betorphal; Meiji Seika Pharma, Co., Ltd.). Tissues were weighed, frozen in liquid nitrogen, and stored at −80°C.

**Table 1:** .Dietary composition (g/kg)

Components	ND	ND + L	HCD	HCD + L
Casein	200	200	200	200
Soybean oil	50	50	10	10
Lard	–	–	50	50
Vitamin mix (AIN‐76)	10	10	10	10
Mineral mix (AIN‐76)	40	40	40	40
Choline hydrogen tartrate	2.0	2.0	2.0	2.0
Cellulose powder	20	20	20	20
Sucrose	100	100	100	100
Corn starch	578	578	578	578
Cholesterol	–	–	5	5
Sodium cholate	–	–	2.5	2.5
*tert‐*Butylhydroquinone	0.014	0.014	0.014	0.014
LP06CC2	–	50	–	50

### 2.3 Biochemical analyses of plasma, adipose tissue, and liver samples

Liver and serum triglyceride (TG), total cholesterol (TC) levels, and serum aspartate aminotransferase (AST) and alanine transaminase (ALT) activities were measured using the TG E‐test, cholesterol E‐test, and transaminase CII‐test (all from Wako Pure Chemical Industries), respectively. The livers (100 mg) were homogenized (4,000 rpm for 3 min at 4°C) with a bead cell disruptor Micro Smash MS‐100R (TOMY SEIKO Co., Ltd.); lipids were extracted with chloroform‐methanol (2:1) and subjected to lipid analysis. The extract was dried under nitrogen gas and re‐dissolved in 2‐propanol containing 5% (v/v) Tween‐20 for TG and TC determination.

### 2.4 Western blotting

The liver was homogenized in 50 mM Tris‐HCl (pH 7.5), 150 mM NaCl, 1 mM EDTA, 50 mM NaF, 30 mM Na_4_P_2_O_7_, and 2% (v/v) Triton X‐100 along with a protease inhibitor cocktail (Nacalai Tesque, Inc.). The protein concentration was determined using the BCA protein assay kit (Thermo Fisher Scientific, Inc.). Further, 10 µg of protein was subjected to SDS‐PAGE using 12% gels and then transferred to a PVDF membrane. Subsequently, the membrane was blocked with Blocking One‐P (Nacalai Tesque, Inc.) for 30 min at 25°C. Next, the membrane was treated with antibodies against cholesterol 7 alpha‐hydroxylase (Cyp7A1) (Abcam plc, ab65596) and β‐actin (clone AC‐15, Sigma). The membrane was washed three times and then treated with the secondary antibodies anti‐rabbit IgG‐HRP or anti‐mouse IgG‐HRP (Cell Signaling Technology) for 1 hr at 25°C. Signals were visualized using the ECL Western blotting substrate (Bio‐Rad). Subsequently, the band intensities were determined using the chemiluminescent imaging system, LAS‐4000 (Fujifilm). The intensity of the bands was normalized using a corresponding β‐actin band as an internal control. The band intensities of Cyp7A1 were normalized with those of β‐actin used as an internal control.

### 2.5 LC/MS analysis of bile acids

Bile acids in feces were measured according to the method of Hagio et al. (2009). Feces were freeze‐dried and ground thoroughly. One milliliter of ethanol was added to 100 mg of the ground samples to extract bile acids. Nordeoxycholic acid (NDCA, 25 nmol) was added as an internal standard to each sample. The samples were subjected to sonication and then heated at 60°C for 30 min in a water bath. The samples were cooled through immersion in cold running water, heated in boiling water for 3 min, and centrifuged at 1,600 *g* for 10 min at 15°C. The supernatants were then collected, and the pellets were washed thrice with ethanol and centrifuged at 11,200 *g* for 1 min to collect supernatants. The pooled extracts were evaporated and resolved into 1 ml of methanol followed by purification using Ultrafree‐MC‐HV centrifugal filter units (Millipore) for LC/MS analysis. Liquid chromatography (LC) separation was conducted using the ACQUITY UPLC H‐class system (Waters) equipped with an ACQUITY UPLC HSS T3 column (1.7 μm, 100 mm × 2.1 mm; Waters) and maintained at 40°C. Solvent A was water containing 0.1% formic acid and solvent B was acetonitrile containing 0.1% formic acid. Elution was conducted with a linearly increasing concentration gradient of acetonitrile at 0.4 ml/ml. The acetonitrile concentration was increased from 25% to 27% for 4 min, from 27% to 35% for 2 min, from 35% to 45% for 9 min, from 45% to 70% for 4 min, and from 70% to 100% for 0.5 min. The *m/z* values for each bile acid are shown in Table S1.

### 2.6 LC/MS analysis of short‐chain fatty acids

Fecal short‐chain fatty acids (SCFA) were determined according to previous reports (Inoue et al., 2019; Miwa et al., 1987). Fecal samples (0.5 g) were suspended in 15 ml of H_2_O and centrifuged at 8,000 *g* for 10 min, and the supernatant used for the determination of SCFA. The supernatant (100 μl) was mixed with 100 μl of the internal compound (500 μM 2‐ethylbutyrate), 200 μl of 20 mM 2‐nitrophenylhydrazine‐HCl (2‐NPH), and 400 μl of 1‐ethyl‐3‐(3‐dimethylaminopropyl) carbodiimide‐HCl, then heated at 60°C for 20 min. Next, 100 μl of 15% potassium hydrochloride in methanol: H_2_O (4:1) was added and heated at 60°C for 15 min. 2‐NPH‐labeled SCFAs were extracted twice with diethylether. Extracts were evaporated and resolved into methanol for the LC/MS analysis. The elution from the ACQUITY UPLC HSS T3 was conducted with a linearly increasing concentration gradient of acetonitrile consisting of water containing 0.05% formic acid (phase A) and acetonitrile containing 0.05% formic acid (phase B) at a flow rate of 0.15 ml/min. The pump was programmed as follows: during the first 5 min 20% phase B, then phase B was increased from 20% to 60% for 15 min and kept for 2 min. A 10 μl sample was injected into the system with the conditioned at 10°C and column temperature maintained at 50°C. The ESI‐MS was operated in a positive and negative ion mode and the capillary voltage was adjusted at 3.0 kV. Lactic (LA), acetic (AA), propionic (PA), *iso*‐butyric (*i*BA), *n*‐butyric (*n*BA), *iso*‐valeric (*i*VA), and *n*‐valeric (*n*VA) acids were determined. The *m/z* values for each SCFA are shown in Table S2.

### 2.7 LAB deconjugation of bile acids

Ten strains of LABs isolated from Mongolian dairy products (2.0 × 10^5^ cfu) were seeded and cultured in MRS broth containing 0.2% glycocholate at 37°C for 20 hr. At the end of culture, the pH was adjusted to 7.0 with 5N NaOH. Then, 1 ml of the supernatant was collected and mixed with 2 ml of ethyl acetate. The solution was vigorously mixed and centrifuged at 2,300 *g* for 20 min at 4°C. The ethyl acetate layer was collected and dried up under a nitrogen stream, then 0.25 ml of 0.01 N NaOH, 0.25 ml of 1% furfural, and 1.5 ml of 16 N H_2_SO_4_ were added, and the reaction proceeded at 65°C for 13 min. Finally, 1.25 ml of acetic acid was added and the concentration of free bile acids was determined measuring the optical density at 660 nm.

### 2.8 Statistical analyses

All data are represented with the mean ± *SE*. All statistical analyses were performed using 4‐steps Excel Statistics (OMS). Post hoc tests were performed after a two‐way ANOVA. When significant interaction (*p* < .05) was detected, the Tukey‐Kramer test was conducted to evaluate the significant differences among dietary groups.

## 3 RESULTS

### 3.1 Growth parameters

LP06CC2 and cholesterol did not affect the body weight (Table 2). Food intake and liver weight were higher in the HCD groups. LP06CC2 did not affect the weight of the empty cecum. The weight of the cecum content is shown in Table 4. The weight of the epididymal fat was significantly lower in the LP06CC2 fed mice. The weight of the renal fat was lower in HCD groups; however, LP06CC2 had no apparent effect.

**Table 2:** Effect of *L. plantarum* 06CC2 on the growth parameters in Balb/c mice fed cholesterol‐loaded diet

	ND	ND + L	HCD	HCD + L
Body weight (g)				
Initial	21.1 ± 0.4	21.0 ± 0.3	21.1 ± 0.4	21.0 ± 0.4
Final	24.1 ± 0.2	24.7 ± 0.6	24.7 ± 0.5	24.5 ± 0.5
Food intake (g/day)	4.0 ± 0.2^a^	4.2 ± 0.2^ab^	4.8 ± 0.2^b^	4.8 ± 0.1^b^
Tissue weight (mg/g body wt.)				
Liver	42.3 ± 1.2^a^	44.2 ± 2.9^a^	68.7 ± 5.2^b^	76.7 ± 6.5^c^
Cecum	1.9 ± 0.3	2.3 ± 0.2	2.2 ± 0.5	2.1 ± 0.2
Epididymal fat	17.0 ± 3.3^a^	13.6 ± 3.8^ab^	13.4 ± 2.3^ab^	10.1 ± 1.2^b^
Renal fat	4.8 ± 1.5^a^	5.3 ± 2.8^a^	1.6 ± 0.6^b^	1.4 ± 0.5^b^

	Two‐way ANOVA					
	Final BW	Food intake	Liver	Cecum	Epididymal fat	Renal fat
Cholesterol	NS	*p* < .01	*p* < .001	NS	*p* < .01	*p* < .001
LP06CC2	NS	NS	*p* < .05	NS	*p* < .01	NS
Interaction	NS	NS	NS	*p = *.086	NS	NS

Note

Data are means ± *SE* for six samples. Post hoc test was performed after two‐way‐ANOVA. When significant interaction was detected Tukey‐Kramer test was done to evaluate the significant different among dietary groups. NS; not significant (*p > *.1). Values not sharing any common superscript letter are significantly different each other.

### 3.2 Lipid parameters

Serum and liver lipid profiles were analyzed and are shown in Table 3. Dietary cholesterol decreased serum TG and increased serum and hepatic cholesterol. Serum TG was significantly decreased by LP06CC2 whereas no interaction was detected. Dietary LP06CC2 significantly decreased the hepatic cholesterol level and the post hoc test revealed a significant reduction in mice fed HCD. Serum AST and ALT activities were measured as indices of hepatic injury. ALT is more specific to hepatic injury and cholesterol feeding significantly but moderately increased its serum activity. LP06CC2 slightly but not significantly suppressed the increase of ALT activity in mice fed HCD.

**Table 3:** Effect of *L plantarum* 06CC2 on the lipid parameters in Balb/c mice fed cholesterol‐loaded diet

	ND	ND + L	HCD	HCD + L
Serum (mg/dl)				
TG	148 ± 11^a^	117 ± 9^ab^	65 ± 5^b^	57 ± ^7b^
Total cholesterol	50 ± 8^a^	61 ± 8^a^	122 ± 11^b^	126 ± 6^b^
Liver (mg/g)				
TG	62 ± 20	49 ± 8	46 ± 7	36 ± 2
Total cholesterol	5.4 ± 0.4^a^	4.6 ± 0.1^a^	64.2 ± 1.6^b^	53.6 ± 2.2^c^
Hepatic injury				
AST (IU)	102 ± 16	145 ± 41	308 ± 86	177 ± 37
ALT (IU)	44 ± 9	43 ± 6	76 ± 10	60 ± 7

	Two‐way ANOVA					
	Serum TG	Serum T‐chol	Liver TG	Liver T‐chol	AST	ALT
Cholesterol	*p* < .001	*p* < .001	NS	*p* < .001	NS	*p* < .01
LP06CC2	*p* < .05	NS	NS	*p* < .001	NS	NS
Interaction	NS	NS	NS	*p* < .01	*p < *.055	NS

Note

Data are means ± *SE* for six samples. Post hoc test was performed after two‐way‐ANOVA. When significant interaction was detected Tukey‐Kramer test was done to evaluate the significant different among dietary groups. NS; not significant (*p > *.1). Values not sharing any common superscript letter are significantly different each other.

### 3.3 Fecal parameters

Dietary cholesterol did not affect the fecal weight whereas LP06CC2 significantly increased it irrespective of the dietary condition (Table 4). In addition, the two‐way ANOVA analysis showed the significant increase of cecal content by cholesterol and LP06CC2 whereas no interaction was detected. Cecum pH was slightly increased by LP06CC2.

**Table 4:** Effect of *L. plantarum* 06CC2 on the fecal parameters in Balb/c mice fed cholesterol‐loaded diet

	ND	ND + L	HCD	HCD + L
Fecal weight (g/day)	2.22 ± 0.13^a^	3.75 ± 0.19^b^	2.60 ± 0.08^a^	3.27 ± 0.09^b^
Cecum content (mg/g body wt.)	5.97 ± 1.32^a^	8.55 ± 0.23^ab^	8.77 ± 0.72^ab^	12.35 ± 0.85^b^
Cecum pH	7.57 ± 0.06^a^	7.84 ± 0.05^ab^	7.79 ± 0.10^ab^	7.86 ± 0.08^b^

	Two‐way ANOVA		
	Fecal weight	Cecum content	Cecum pH
Cholesterol	NS	*p* < .01	NS
LP06CC2	*p* < .001	*p* < .01	*p* < .05
Interaction	*p* < .01	NS	NS

Note

Data are means ± *SE* for six samples. Post hoc test was performed after two‐way‐ANOVA. When significant interaction was detected Tukey‐Kramer test was done to evaluate the significant different among dietary groups. NS; not significant (*p > *.1). Values not sharing any common superscript letter are significantly different each other.

### 3.4 Bile acid analysis

**Figure 1:**
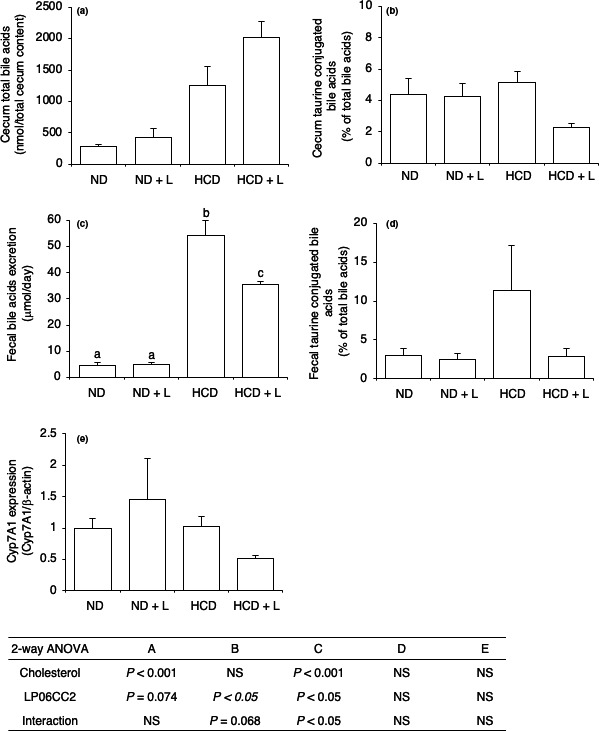
Effect of *Lactobacillus plantarum* 06CC2 on the excretion, deconjugation, and biosynthesis of bile acids in mice fed a high‐cholesterol diet. (a) Cecal total bile acids, (b) Cecal conjugated bile acid level shown as taurine‐conjugated fatty acids, (c) Daily fecal bile acid excretion, (d) Fecal conjugated bile acid level shown as taurine‐conjugated fatty acids, (e) Hepatic Cyp7A1 expression shown as relative to β‐actin. Data are means ± *SE* for six mice. Post hoc test was performed after a two‐way ANOVA. When a significant interaction was detected, the Tukey‐Kramer test was conducted to evaluate the significant differences among dietary groups. NS, not significant (*p* > .1)

Cecal and fecal bile acid compositions were analyzed using LC/MS to evaluate the effect of LP06CC2 on the bile acid metabolism. First, HCD increased the cecal total bile acids and the fecal bile acid excretion. Under cholesterol‐loaded condition, LP06CC2 increased the cecal total bile acid whereas it decreased the fecal bile acid excretion (Figure 1a,c). To evaluate the effect on bile acid deconjugation, the taurine‐conjugated bile acid content was calculated and is shown in Figure 1b (cecum) and 1D (feces). Cholic acid, α‐muricholic acid, β‐muricholic acid, chenodeoxycholic acid, deoxycholic acid, lithocholate, hyodeoxycholic acid, ursodeoxycholate (UDCA), and their taurine conjugate form were detected, except for UDCA (data no shown). LP06CC2 significantly decreased the percentage of cecal conjugated bile acid level under cholesterol‐loaded conditions. The percentage of fecal conjugated bile acids was also decreased by LP06CC2 under cholesterol‐loaded conditions although the difference was not significant. The protein expression of Cyp7A1, a rate‐limiting enzyme for the synthesis of bile acids from cholesterol in the liver, was measured as bile acid levels were modulated by LP06CC2 (Figure 1e). Cyp7A1 protein expression was slightly but not significantly decreased by LP06CC2 in mice fed HCD.

### 3.5 Bile acid deconjugation in vitro

We isolated several LABs from Mongolian dairy products and selected some of them that were estimated to have probiotic properties. Here, we evaluated their ability to deconjugate bile acids in vitro (Table 5) using glycocholate. The deconjugation ability is shown as deconjugation (%) of conjugated bile acid in the MRS medium. The results ranged from 1.50% to 20.44% and LP06CC2 was the strain that showed the most potent ability to deconjugate among the LABs tested.

**Table 5:** Deconjugation of bile acid by several lactic acid bacteria

LB	Free cholic acid (μmol/L)	Deconjugation (%)
*Lactobacillus plantarum* 06CC2	2.04 ± 0.66	20.44 ± 6.58
*Lactobacillus plantarum* 05AM23	0.82 ± 0.03	8.24 ± 0.34
*Lactobacillus plantarum* 06TCa8	1.24 ± 0.33	12.44 ± 3.26
*Lactobacillus paracasei* ssp. *paracasei* 06TCa19	0.73 ± 0.09	7.33 ± 0.92
*Lactobacillus paracasei* ssp. *paracasei* 06TCa22	0.44 ± 0.26	4.40 ± 2.63
*Lactobacillus paracasei* ssp. *tolerans* 06TCa39	0.19 ± 0.17	1.93 ± 1.69
*Lactobacillus plantarum* 06TCa40	1.04 ± 0.75	10.44 ± 7.51
*Lactobacillus paracasei* ssp. *paracasei* 06TCa43	0.29 ± 0.01	2.90 ± 0.12
*Lactobacillus delbrueckii* ssp. *lactis* 06TC3	0.15 ± 0.13	1.50 ± 1.35
*Lactobaillus plantarum* 06CC9	0.67 ± 0.23	6.74 ± 2.26

Note

Data are means ± *SE* for three experiments.

### 3.6 Fecal short‐chain fatty acids analysis

**Figure 2:**
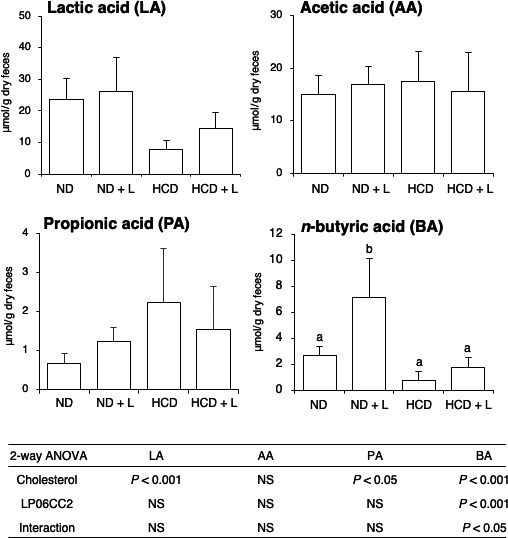
Effect of *Lactobacillus plantarum* 06CC2 on the fecal short‐chain fatty acid levels in mice fed a high‐cholesterol diet. Results are shown as μmol/g dry feces. Data are means ± *SE* for six mice. Post hoc test was performed after a two‐way‐ANOVA. When a significant interaction was detected, the Tukey‐Kramer test was conducted to evaluate the significant differences among dietary groups. NS: not significant (*p* > .1)

Fecal SCFA levels were measured through LC/MS. LA and PA levels were significantly modulated by HCD (Figure 2). Although LP06CC2 did not regulate fecal LA, AA, and PA levels, the *n*BA level was increased by LP06CC2. The increase of *n*BA by LP06CC2 was notably observed in mice fed the ND + L diet compared with mice fed the ND diet and moderately observed in mice fed cholesterol‐loaded diets. *i*BA and *i*VA were not detected in any sample and *n*VA was detected in 4/6 samples in ND + L group (0.07–0.17 mmol/g dry feces). The Pearson's correlation coefficient analysis revealed a strong and significant positive correlation between fecal LA and *n*BA, and between AA and *n*BA.

## 4 DISCUSSION

In this contribution, we evaluated the effect of LP06CC2 on the cholesterol metabolism in mice fed a cholesterol‐loaded diet. Notable findings of this study were the reduction of the hepatic cholesterol via promotion of the bile acid deconjugation and the increase of fecal nBA.

Several reports focus on the bile acid hydrolase activity since the enzyme is one of the putative targets for the cholesterol‐reducing effect of LAB. Bile acid hydrolase recognizes bile acids on both the cholate steroid nucleus and the amino acid groups (glycine/taurine) and catalyzes the deconjugation. Bile acid deconjugation results in a decrease of their intestinal absorption because conjugated bile acids can be selectively absorbed via several transporters to the liver ( Dawson & Karpen, 2015). Therefore, the manipulation of bile acid deconjugation reflects in the percentage of cecal deconjugated bile acids (Ovadia et al., 2019). Data show that LP06CC2 tended to increase the cecal total bile acid level whereas significantly decreased the percentage of deconjugated bile acids indicating its substantial deconjugation effect. This result may suggest that LP06CC2 prevented the absorption of bile acids in the small intestinal leading to a reduction in the hepatic cholesterol level. Another possible mechanism is the increase of bacterial flora having bile acid hydrolase activity. Although we have not investigated the metagenomics of the intestinal microflora of the mice fed LP06CC2, a variety of resident intestinal microflora have substantial ability to deconjugate bile acids (Jones et al., 2008; Ridlon et al., 2014), and the increase of deconjugation by the ingestion of *L. plantarum* H6 strain is explained by the manipulation of this microflora (Qu et al., 2020). Further studies are needed to understand the detailed mechanism for the LP06CC2 direct or indirect manipulation of microflora. The composition of primary bile acids is somewhat different between mice and humans, and α,β‐muricholic acid, which is rarely detected in humans, was detected in this and our previous studies (Kosakai et al., 2019). On the other hand, the conjugated bile acids are reabsorbed in the small intestine and the free bile acids are metabolized to secondary bile acids in the lower gastrointestinal tract in mice and humans. Therefore, the cholesterol‐lowering effect of LP06CC2 through bile acid deconjugation is expected to be exerted in humans. On the other hand, because the composition of the entire gut microbiota and the degree LP06CC2 occupancy may affect this effect, further studies on the gut microbiota analysis and the most appropriate dosage are needed.

Although the reduction of taurine‐conjugated bile acids was observed in the LP06CC2 ingestion, the total bile acid excretion was significantly reduced. In addition, the expression of Cyp7A1, a rate‐limiting enzyme of the bile acid synthesis from cholesterol, was also suppressed. As observed in Cyp7A1‐deficient mice, the fecal bile acid level was lower than that in wild‐type mice (Erickson et al., 2003). Therefore, the expression of hepatic Cyp7A1 protein reflects the hepatic cholesterol level and the influx of bile acid from the enterohepatic circulation (Chiang, 2015). In contrast, several reports showed that LAB ingestion promotes hepatic Cyp7A1 protein expression together with an increase of the efflux as bile acids (Heo et al., 2018; Hu et al., 2013; Qu et al., 2020) It seems that such cholesterol‐reducing mechanism did not occur in this study.

The increase of the cecal content is promoted through the intestinal fermentation of dietary fiber and is often accompanied with an increase of SCFAs such as AA, PA, and *n*BA (Berggren et al., 1995; Besten et al., 2013). Some of the health beneficial effects of probiotics and dietary fibers may be explained via the increase of intestinal SCFAs. Here, our data revealed that LP06CC2 specifically increased fecal *n*BA but no other SCFAs indicating stimulation of *n*BA bacteria such as *Clostridium* cluster IV, XIVa (Barcenilla et al., 2000; Pryde et al., 2002). Notably, LP06CC2 failed to increase fecal LA even though it is considered a probiotic strain. Recent studies have uncovered the importance of LA as the main substrate for *n*BA synthesis by butyrate‐producing intestinal bacteria such as *Clostridium* XIVa (Bourriaud et al., 2005; Duncan et al., 2004). As observed in Figure 2, fecal LA and *n*BA levels showed a similar trend of being downregulated by cholesterol and increased by LP06CC2. Moreover, the Pearson’s correlation coefficient analysis between fecal LA and *n*BA showed a strong and significant correlation (*r* = .8206, *p* < .01, Figure S1). This finding indicates that the intestinal LA supply may be an important factor for the manipulation of BA production.

Generally, SCFAs regulate cholesterol metabolism‐related gene expression leading to the production of SREBP2, LDL receptor, and Cyp7A1, and results in the reduction of the cholesterol level (Zhao et al., 2017). This mechanism for the regulation of cholesterol levels may not be appropriate for LP06CC2 in that the increase of fecal AA and PA levels and hepatic Cyp7A1 expression was not observed. Notably, *n*BA may reduce cholesterol absorption via downregulation of the Niemann‐Pick C1‐Like 1 (NPC1L1) and upregulation of ABCG5 and G8 expression (Chen et al., 2018; Nguyen et al., 2019).^.^ In addition, some reports reveal that LAB directly regulates NPC1L1 and results in the suppression of cholesterol absorption in vitro (Le & Yang, 2019; Lim et al., 2017). NPC1L1 is a cholesterol transporter and regulates the whole body cholesterol pool and NPC1L1 knockout mice resistance to diet‐induced hepatic cholesterol increase (Davis et al., 2004). Therefore, further studies are needed to evaluate the putative mechanism via regulation of the intestinal cholesterol transporter including NPC1L1.

Unexpectedly but intriguingly, serum TG and epididymal fat weight were significantly decreased by LP06CC2. We have not evaluated the detailed mechanism underlying these observations but it may be important for the application of LP06CC2 to prevent obesity and hyperlipidemia as shown in other strains of *L. plantarum* (Choi et al., 2020; Wu et al., 2015). Nguyen et al. (2019) suggested a possible mechanism where nBA reduces hepatic cholesterol and triglyceride via regulation of hepatic PPARα through SCFA receptors on the intestinal cell. This hypothesis is supported by our results which also show the reduction of both hepatic cholesterol and serum triglyceride by LP06CC2.

## CONFLICT OF INTEREST

We declare no conflict of interest associate with this manuscript.

## AUTHOR'S CONTRIBUTION

Masao Yamasaki, Chuluunbat Tsend‐Ayush, Tsendesuren Oyunsuren, Yiran Li, Tomoki Nakano, Masahiko Takeshita, and Yuo Arima wrote the manuscript, designed the experiments, contributed to data collection, and critically reviewed the manuscript. Mikako Minesaki, Yuko Miyamoto, Asuka Iwakiri, Kenjiro Ogawa, and Kazuo Nishiyama contributed to the analysis and interpretation of data and assisted in the preparation of the manuscript. All authors equally contributed to this work and approved the final version of the manuscript.

## Supporting information

Fig S1

Table S1‐S2
