# Supplementary figures and images for: Lactobacillus plantarum 06CC2 reduces hepatic cholesterol levels and modulates bile acid deconjugation in Balb/c mice fed a high‐cholesterol diet
Source: Food Sci Nutr. 2020 Oct 26;8(11):6164–73. doi: 10.1002/fsn3.1909 (PMC7684586; doi:10.1002/fsn3.1909)

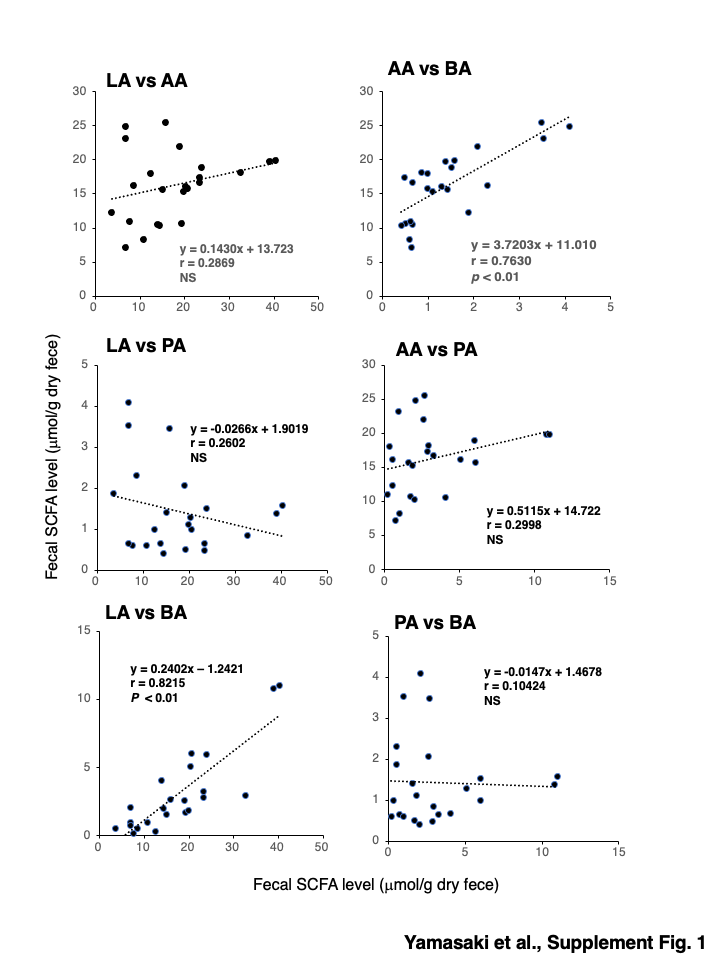

Supplement: Supplementary file 1 — Fig S1 [file FSN3-8-6164-s001.tiff]
