# Supplementary material for: Lactobacillus plantarum 06CC2 reduces hepatic cholesterol levels and modulates bile acid deconjugation in Balb/c mice fed a high‐cholesterol diet
Source: Food Sci Nutr. 2020 Oct 26;8(11):6164–73. doi: 10.1002/fsn3.1909 (PMC7684586; doi:10.1002/fsn3.1909)
Supplement: Supplementary file 2 — Table S1‐S2 [file FSN3-8-6164-s002.docx]

Table S1 The *m/z* values of product ions from bile acids.

| Compound | | *m/z* values |
| --- | --- | --- |
| Cholic acid | CA | 407.3 |
| α-Murichoric acid | αMCA | 453.3 |
| β- Murichoric acid | βMCA | 453.3 |
| Chenodeoxycholic acid | CDCA | 437.3 |
| Deoxycholic acid | DCA | 391.3 |
| Lithocolic acid | LCA | 421.3 |
| Hyodeoxychoric acid | HDCA | 437.3 |
| Ursodeoxycholic acid | UDCA | 437.3 |
| Taurocholic acid sodium salt hydrate | T-CA | 514.3 |
| Taurodeoxycholic acid sodium hydrate | T-DCA | 498.3 |
| Taurolithocolic acid | T-LCA | 482.3 |
| Taurochenodeoxycholic acid | T-CDCA | 498.3 |
| Taurohyodeoxychoric acid | T-HDCA | 498.3 |
| Tauro-α-Murichoric acid | T-αMCA | 514.3 |
| Tauro-β- Murichoric acid | T-βMCA | 514.3 |
| Nordeoxycholic acid | NDCA | 377.3 |

Table S2 The *m/z* values of product ions from short chain fatty acids.

| Compound | *m/z* values |
| --- | --- |
| Lactic acid | 225.95 |
| Acetic acid | 195.94 |
| Propionic acid | 209.97 |
| *iso* - butyric acid | 223.97 |
| *n* - butyric acid | 223.97 |
| *iso* - valeric acid | 238.01 |
| *n* - valeric acid | 238.01 |
| 2-ethylbutyric acid | 252.01 |
